# Episodic Binge-like Ethanol Reduces Skeletal Muscle Strength Associated with Atrophy, Fibrosis, and Inflammation in Young Rats

**DOI:** 10.3390/ijms24021655

**Published:** 2023-01-14

**Authors:** Constanza Cáceres-Ayala, Rodrigo G. Mira, María José Acuña, Enrique Brandan, Waldo Cerpa, Daniela L. Rebolledo

**Affiliations:** 1 Centro de Excelencia en Biomedicina de Magallanes (CEBIMA), Universidad de Magallanes, Punta Arenas 6213515, Chile; 2 Laboratorio de Función y Patología Neuronal, Departamento de Biología Celular y Molecular, Facultad de Ciencias Biológicas, Pontificia Universidad Católica de Chile, Santiago 8331150, Chile; 3 Centro Integrativo de Biología y Química Aplicada (CIBQA), Universidad Bernardo O’Higgins, Santiago 8370854, Chile; 4 Centro Científico y Tecnológico de Excelencia Ciencia & Vida, Santiago 7780272, Chile; 5 Facultad de Medicina y Ciencia, Universidad San Sebastián, Santiago 7510157, Chile; 6 Centro de Envejecimiento y Regeneración (CARE), Facultad de Ciencias Biológicas, Pontificia Universidad Católica de Chile, Santiago 8331150, Chile

**Keywords:** binge-drinking, alcohol, ethanol, skeletal muscle, muscle fatigue, fibrosis, atrophy, CCN2/CTGF, alcoholic myopathy

## Abstract

Binge Drinking (BD) corresponds to episodes of ingestion of large amounts of ethanol in a short time, typically ≤2 h. BD occurs across all populations, but young and sports-related people are especially vulnerable. However, the short- and long-term effects of episodic BD on skeletal muscle function have been poorly explored. Young rats were randomized into two groups: control and episodic Binge-Like ethanol protocol (BEP) (ethanol 3 g/kg IP, 4 episodes of 2-days ON-2-days OFF paradigm). Muscle function was evaluated two weeks after the last BEP episode. We found that rats exposed to BEP presented decreased muscle strength and increased fatigability, compared with control animals. Furthermore, we observed that skeletal muscle from rats exposed to BEP presented muscle atrophy, evidenced by reduced fiber size and increased expression of atrophic genes. We also observed that BEP induced fibrotic and inflammation markers, accompanied by mislocalization of nNOSµ and high levels of protein nitration. Our findings suggest that episodic binge-like ethanol exposure alters contractile capacity and increases fatigue by mechanisms involving atrophy, fibrosis, and inflammation, which remain for at least two weeks after ethanol clearance. These pathological features are common to several neuromuscular diseases and might affect muscle performance and health in the long term.

## 1. Introduction

Alcohol is the most used drug worldwide, and the occurrence of problematic drinking is worrying. According to the World Health Organization (WHO), 5% of all deaths are attributable to problematic alcohol consumption, which rises to 13.5% in people aged 20–39 years old. These deaths are the consequence of interpersonal violence and domestic traffic and accidents, but also because of the consequent risk factor for multiple chronic diseases [1,2].

The addictive effects of alcohol have been widely studied, as well as the consequences of chronic consumption over time. However, chronic ethanol drinking, associated with alcohol use disorder and dependence, only includes a part of the population that consumes alcohol. Additionally, a different pattern of problematic consumption is called binge drinking (BD), which is not necessarily associated with dependence. BD, also called episodic heavy drinking, occurs mainly during leisure time or on weekends and repeats after periods of detoxification and abstinence [3,4]. BD leads to a quick rise in blood alcohol concentration, reaching 0.8 g/L and more [5], and it is characterized by episodes of ingestion of large amounts of alcohol in a short time, typically less than 2 h (approximately five drinks/men and four drinks/women, considering a standard drink containing 14 g of pure alcohol) [4]. Contrary to chronic dependent consumption, the risk perception for BD consequences in human health is low, especially in the long term, which encourages consumption [6,7,8]. Hence, BD affects a large population, especially adolescents and young people, and has severe negative consequences [9,10,11,12]. Furthermore, BD is highly prevalent in amateur and professional sports, especially in male group sports, where this conduct emerges as a way to cope with the stress of competition and the need to fit into a social structure [13,14,15,16,17].

Alcoholic myopathy, characterized by skeletal muscle weakness and atrophy, affects many consumers with a history of chronic alcohol abuse [18,19,20]. Clinical studies and preclinical laboratory research have led to an understanding of some of the mechanisms involved in alcoholic myopathy associated with chronic consumption, such as alteration in anabolic and catabolic pathways, impaired regeneration, increased inflammation and fibrotic markers, and deficiencies in mitochondrial function leading to energy imbalance and increased oxidative stress [14,19,21,22]. Nevertheless, whether similar, or other pathological mechanisms occur as a result of episodic BD has been less explored. For example, most studies in animal models use chronic or acute (single dose) ethanol exposure, whereas investigations in humans concentrate on the history of chronic consumers or perform protocols of acute ethanol administration, usually below BD doses [14,18,19,21,23]. Therefore, episodic BD, the most common consumption pattern in youth and sportspeople, is under-represented in all these studies. Consequently, little is known about the short- and long-term effects of BD on skeletal muscle function, which might affect athletic performance and quality of life.

In the last few years, a few studies in murine models have evaluated contractile properties under ethanol doses equivalent to BD. However, the BD model consisted of a single administration in those studies, and contractile function was measured 2 h [24], or 1 and 24 h [25] after ethanol administration. Therefore, cellular and molecular alterations leading to functional damage upon ethanol consumption/administration that might take longer to develop would be unnoticed from those observations. Furthermore, BD in humans, especially during youth, corresponds to episodic repetitive events between abstinent periods, which was not addressed in those studies or others. In the present work, we performed a Binge-like Ethanol Protocol (BEP) in young rats designed to mimic intermittent episodic BD [26]. Using this model, members of our group previously showed BEP-driven alterations in brain function, including impairment in cognitive tasks, increased neuroinflammation, oxidative stress, and mitochondrial dysfunction [27,28,29]. Here we evaluated the skeletal muscle contractile properties and possible pathological markers two weeks after the last BEP episode, aiming to examine alterations that can persist in time and contribute to skeletal muscle dysfunction due to episodic BD. Our findings suggest that episodic BEP decreases muscle strength and increases fatigability, associated with the establishment of muscle atrophy and fibrosis, inflammation, and nitrosative stress.

## 2. Results

### 2.1. Repetitive Binge-like Ethanol Administration Decreases TA Muscle Strength and Increases Muscle Fatigability

**Figure 1:**
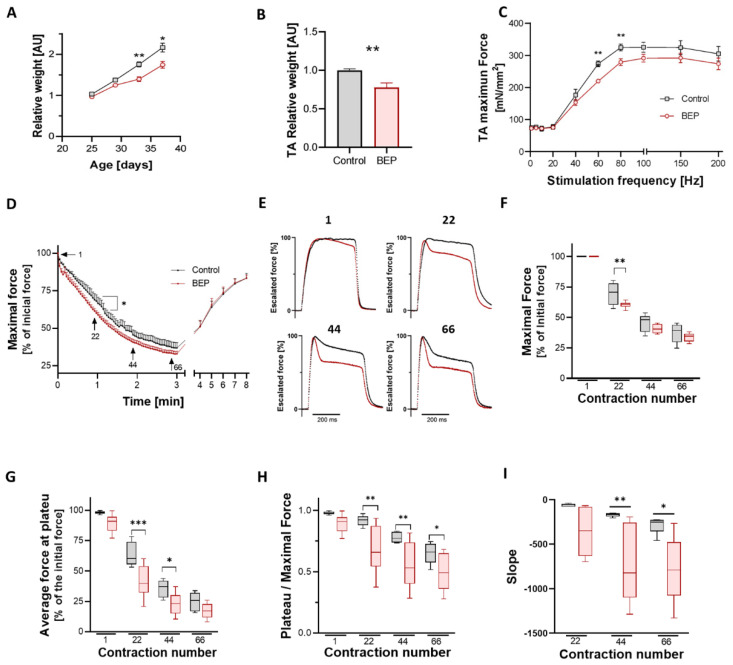
Binge-like ethanol protocol decreased TA force and increased muscle fatigability. (**A**) Relative weight gain, calculated as a fraction of weight in day 1 (PND 25), and relativized to control animals. Control in black N = 8 and BEP in red N = 8. (**B**) Relative weight of TA from BEP-treated (N = 6) vs. control (N = 5) rats. Graphs represent mean ± SEM. (**C**) TA specific force values at various stimulation frequencies for each group: control (N = 7) and BEP-treated (N = 8) rats. (**D**) Fatigue protocol. For each time point, the maximal force from tetanic contraction was normalized to the initial maximal force. Control in black, N = 5; BEP in red, N = 6. (**E**) Profiles from tetanic contractions from the fatigue protocol in (**D**) (correspond to contractions N° 1, 22, 44 and 66). Each profile was escalated to 100%, corresponding to that specific contraction’s maximal force, and the group’s average was plotted. BEP-group shows a more pronounced force decline during tetanic contraction. (**F**) Comparison of maximal force for sampled contractions of the fatigue protocol. (**G**) Comparison of the average force at the last 50 ms of tetanus (plateau) in the sampled contractions of the fatigue protocol. (**H**) Ratio between the plateau and maximal force, as a measure of force decline in sampled contractions. (**I**) The velocity of the force fall was evaluated using the slope of the curve 50 ms after the peak force. For graphs (**F**–**I**), data were analyzed using two-way ANOVA with Fisher’s LSD multi-comparison test. Except for box and whiskers graphs, data represent mean ± SEM. *p*-values: * *p* ≤ 0.05; ** *p* ≤ 0.01; *** *p* ≤ 0.001.

Juvenile rats (25 days old) were randomly assigned to the control (saline IP injection) or the Binge-like Ethanol Protocol (BEP, 3 g/kg ethanol via IP) groups. We monitored the weight of control and BEP-treated rats during the first day of each injection session. While all individuals had similar weights at the beginning of the protocol, we observed that the relative weight gain of the BEP group was lower than control animals (Figure 1A). At the end of the protocol, we compared the mass of isolated tibial anterior (TA) from both experimental groups, observing that TAs from BEP-treated rats had significatively lower mass than TAs from control rats (Figure 1B). Other muscles were evaluated, and we found differences in EDL but not gastrocnemius and soleus (Supplementary Materials Figure S1).

Two weeks after the last binge-like ethanol administration, we evaluated the contractile properties of the TA muscle using *in situ* approximation, which maintained muscle irrigation and innervation, as described before [30]. After determining the optimum muscle length, the maximal isometric force of the TA muscle was measured by stimulating the sciatic nerve at increasing frequencies. We observed that specific TA force (force normalized to the cross-sectional area) decreased in rats from the BEP group, being statistically different when stimulating at 60–80 Hz (Figure 1C).

Next, we evaluated muscle fatigability by repetitive tetanic contraction of the TA muscle over 3 min. We plotted the maximal isometric force, normalized to the initial force, in the function of time. We observed increased fatigability of rats in the BEP group, which was more evident and statistically significant around the first minute of the protocol (Figure 1D). We observed that the profile of consequent tetanic contractions varied during the fatigue protocol, especially in the BEP group. To illustrate this observation, we sampled four specific contractions along the 3 min of repetitive stimulation (contraction N° 1, 22, 44, and 66). Then, we escalated them to 100% peak force to compare the profile between the groups (Figure 1E). Comparing these escalated profiles, we observed a decline in muscle force during the tetanic contraction, which was more pronounced while more advanced in the protocol (intratetanic fatigue). Furthermore, we observed that the intratetanic force decline was more significant in the BEP group than in control rats. Hence, although the maximal force was not relatively different between control and BEP rats (only in contraction N° 22) (Figure 1F), the average force in the final plateau was significatively lower in the BEP group (Figure 1G). Furthermore, the ratio between the plateau and maximal strength also decreased in BEP-treated rats compared to controls (Figure 1H), with a more pronounced downfall slope (Figure 1I), suggesting that intratetanic muscle force decayed more, and faster, after each contraction.

### 2.2. Repetitive Binge-like Ethanol Administration Causes a Reduction in Muscle Fiber Size

**Figure 2:**
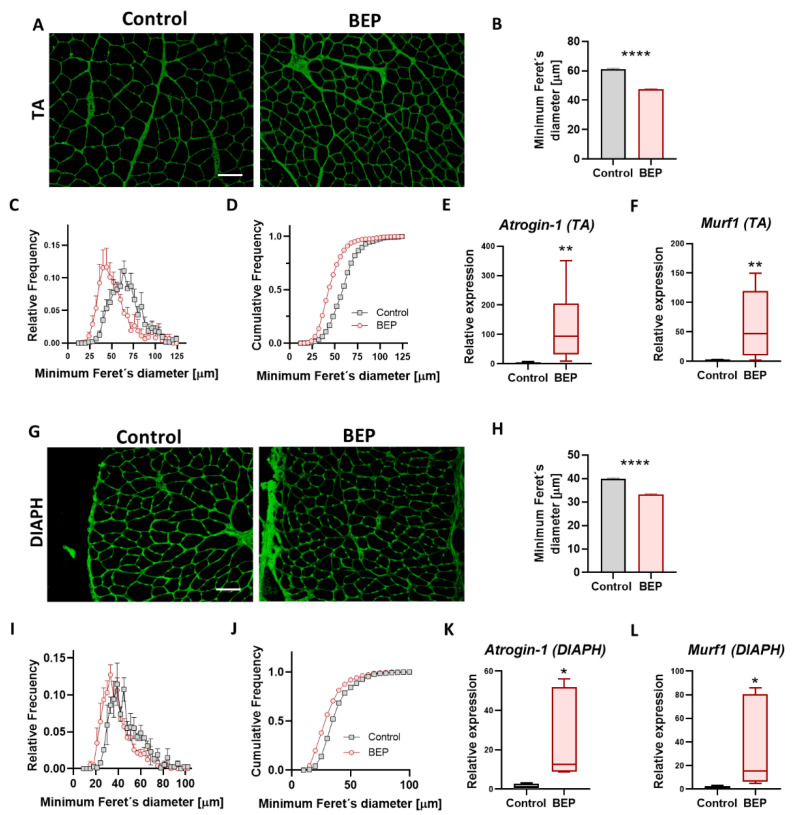
Binge-like ethanol protocol induced skeletal muscle atrophy. (**A**,**G**) Representative images of WGA (green) stained muscle sections of TA (**A**) and DIAPH (**G**). Scale bar 100 µm. (**B**,**H**) Quantification of average minimum Feret’s diameter of skeletal muscle fibers of TA (**B**) and DIAPH (**H**). For TA, Control N = 5, BEP N = 6. For DIAPH, Control N = 2, BEP N = 4. (**C**,**I**) Histograms for relative frequencies of minimum Feret’s diameter in TA (**C**) and DIAPH (**I**). (**D**,**J**) Histograms for cumulative frequencies of minimum Feret’s diameter in TA (**D**) and DIAPH (**J**). (**E**,**K**) Relative mRNA expression of atrophy marker *Atrogin-1* (**E**, TA; **L**, DIAPH). (**F**,**L**) Relative mRNA expression of atrophy marker *Murf1* (**F**, TA; **M**, DIAPH). TA, Control N = 3, BEP N = 4. DIAPH, Control N = 3, BEP N = 3. *p*-values: * *p* ≤ 0.05; ** *p* ≤ 0.01; **** *p* ≤ 0.0001.

A possible mechanism related to decreased muscle force and increased fatigability is muscle waste or atrophy. Alcoholic myopathy is characterized by atrophy, predominantly of fast type 2 glycolytic fibers and type 1 oxidative fibers [19,20,31]. Furthermore, skeletal muscle atrophy is a hallmark of neurodegenerative conditions affecting motor neurons and diseases like cachexia, sarcopenia, and muscle denervation [32,33,34,35,36]. As mentioned before, we observed decreased muscle mass of TA (the muscle in which we measured contractile function) from BEP-treated rats (Figure 1B). We performed basic histology staining (H&E), and we did not find evidence of necrotic foci or central nuclei in muscles from BEP treated rats (Supplementary Materials Figure S2). To evaluate possible muscle atrophy, we used a fluorescent probe (wheat germ agglutinin, WGA) to label cell surfaces in muscle cross-sections [37] and to estimate muscle fiber size. We observed smaller-sized fibers in TA from BEP-treated (Figure 2A), and we evaluated differences in the fiber cross-sectional area by determining the minimum Feret’s diameter. We found that the average fiber size in TA muscles from BEP-treated rats was reduced, compared to controls (Figure 2B). Furthermore, the distribution profile, relative and cumulative, presented a curve displaced to the left with a higher proportion of smaller diameters than the control animals (Figure 2C,D). The reduction in minimum Feret’s diameter was also observed in diaphragm (DIAPH) (Figure 2G–J) and extensor digitorum longus (EDL) muscles (Supplementary Materials Figure S3) of BEP-treated rats.

To evaluate if known mechanisms leading to muscle atrophy were involved in reducing fiber diameter after BEP, we tested for the mRNA levels of *Murf-1* and *Atrogin-1*, E3-ubiquitin ligases that drive protein degradation and that can lead to muscle atrophy [35,38,39,40,41,42,43]. We found elevated expression of *Murf-1* and *Atrogin-1* mRNAs in TA and DIAPH from BEP-treated rats (Figure 2E,F,K,L). However, we did not find significant changes in *Atrogin-1* protein levels (Supplementary Materials Figure S3A). These findings suggested that reduced muscle mass and atrophy could contribute to BEP-induced decline in muscle force and increased fatigue.

Skeletal muscle is a heterogeneous tissue composed of a great diversity of slow and fast fibers, which are versatile and plastic in response to functional and environmental requirements [44]. Change in the type of fiber distribution occurs as an adaptation to different conditions, such as atrophy and exercise [45,46,47]. Then, we evaluated whether increased fatigue in BEP-treated rats could be paired with a changed proportion of slow and fast fibers, using an antibody that recognizes type I skeletal muscle fibers by detecting the slow myosin heavy chain. We found no changes in the percentage of immuno-stained Type I (slow) and non-stained (fast) fibers between control and BEP-treated rats in TA muscle and diaphragm (DIAPH), a muscle much more mixed in terms of the proportion of slow and fast fibers than TA, which is mainly fast [48] (Supplementary Materials Figure S4). Furthermore, a preliminary evaluation in a small group of individuals suggested that the reduction in fiber size was independent of fiber type, and that slow type I, and type IIA fibers had decreased Feret’s diameter (Supplementary Materials Figure S5A–F). Nevertheless, more studies are needed to fully elucidate the contribution of different fiber types in skeletal muscle dysfunction due to binge alcohol.

### 2.3. Exposure to Repetitive Binge-like Ethanol Leads to a Fibrotic Phenotype

**Figure 3:**
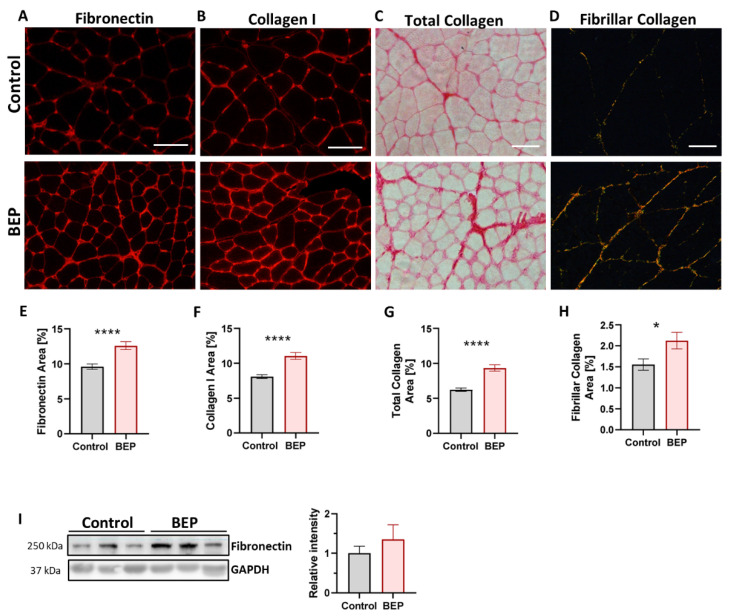
Binge-like ethanol protocol induced ECM proteins accumulation. (**A**–**D**) Representative images of TA immunofluorescence using anti-fibronectin (**A**) and anti-collagen I (**B**) antibodies, and for Sirius Red staining in brightfield microscopy showing total collagen (**C**) and polarized light microscopy showing fibrillar collagen (**D**). Scale bar 100 µm. (**E**–**H**) Quantification of fibronectin (**E**), collagen I (**F**), total collagen (**G**), and fibrillar collagen (**H**) as a percentage of occupied area fraction. Control N = 5, BEP N = 6. (**I**) Immunoblot against fibronectin and GAPDH as a loading control, with the respective densitometric quantification. Control N = 4, BEP N = 4. *p*-values: * *p* ≤ 0.05; **** *p* ≤ 0.0001.

Skeletal muscle fibrosis is the excessive accumulation of extracellular matrix (ECM) proteins around muscle fibers, negatively impacting muscle contractibility, cellular signaling, vascularization, and innervation, among others [49,50,51]. Fibrosis is characteristic of many neuromuscular pathologies of different etiologies, usually directly proportional to disease severity [49,52,53,54]. Thus, the establishment of fibrosis is usually a sign of chronic muscle damage. Therefore, we evaluated if BEP was able to induce a fibrotic process that was still evident two weeks after the last ethanol administration. We used transversal cryosections of skeletal muscle to evaluate levels of ECM proteins, fibronectin, an excellent marker of skeletal muscle fibrosis [36,37,55], and collagen I, by immuno-staining and total collagen using picrosirius-red staining. Fibrillar collagen was evaluated by observing picrosirius-red-stained slides under polarized light [56]. We found that the TA muscle from BEP-treated rats had an augmented accumulation of fibronectin (Figure 3A,E), collagen I (Figure 3B,F), total collagen (Figure 3C,G), and fibrillar collagen (Figure 3D,H), compared to control individuals. The increased levels of fibronectin in TA from BEP-treated rats were also observed using western blot from protein extracts from TA samples, yet we obtained high variability in those samples (Figure 3I). The accumulation of ECM proteins was also evaluated in DIAPH, because DIAPH muscle has been shown to be especially susceptible to fibrosis, more so than other limb muscles, in murine models for neuromuscular diseases [57,58,59]. We found that the increment in ECM accumulation after BEP was also observed in DIAPH (Supplementary Materials Figure S6) and other hindlimb muscles different from TA (Supplementary Materials Figure S7).

**Figure 4:**
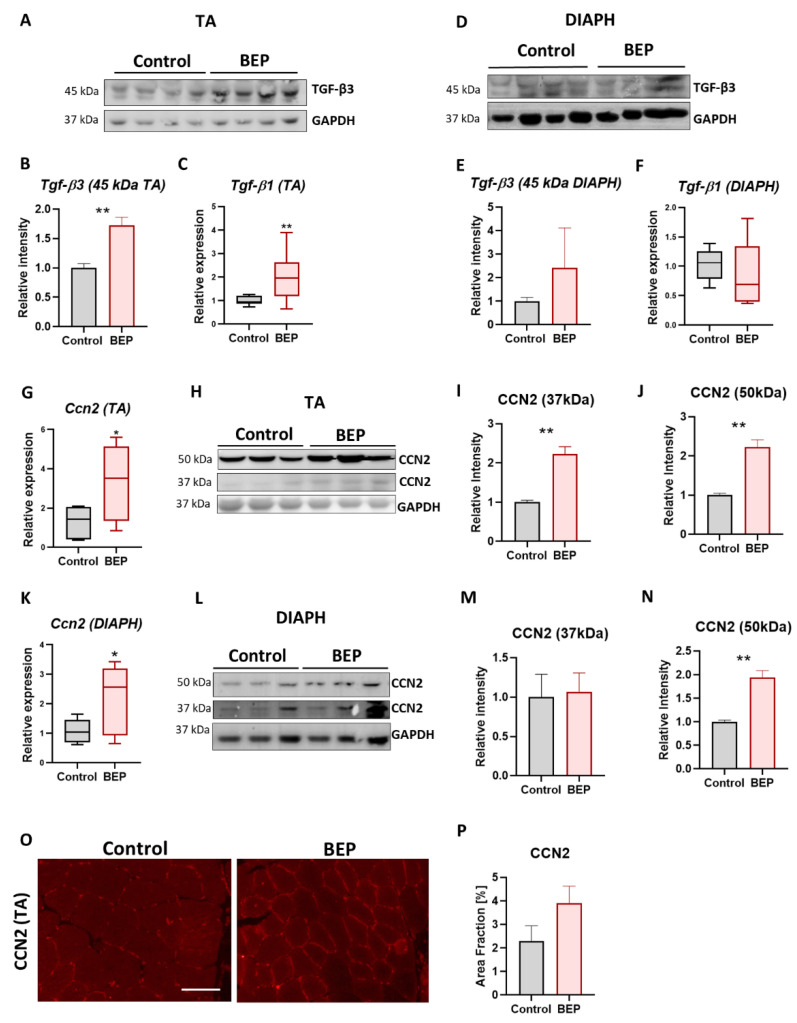
Binge-like ethanol protocol induced profibrotic factors TGF-β and CCN2/CTGF. TGF-β expression in TA muscle. TGF-β3 protein levels with the respective densitometric analysis (**A**,**B**). Control N = 6, BEP N = 4. Relative mRNA levels of *TGF-β1* (**C**). Control N = 3, BEP N = 4. *TGF-β* expression in DIAPH muscle. TGF-β3 protein levels with the respective densitometric analysis (**D**,**E**). Control N = 4, BEP N = 3. Relative mRNA levels of TGF-β1 (**F**). Control N = 3, BEP N = 4. Relative mRNA levels of *Ccn2/Ctgf* in TA (**G**) and DIAPH (**K**). (**H**,**L**) Immunoblot against CCN2/CTGF and GAPDH as a loading control, with the respective densitometric analysis of the 37 and 50 kDa immunoreactive bands. WB performed from whole muscle extracts from TA (**H**–**J**) and diaphragm (**L**–**N**). Control N = 4, BEP N = 4. (**O**) Representative images of TA immunostaining using anti-CCN2/CTGF antibody. Scale bar 100 µm. (**P**) Quantification of CCN2/CTFG as a percentage of occupied area fraction. Control N = 3, BEP N = 4. *p*-values: * *p* ≤ 0.05; ** *p* ≤ 0.01.

Cellular Communication Network Factor 2 (CCN2, also called Connective Tissue Growth Factor CTGF), is a potent profibrotic factor in skeletal muscle and other tissues and a downstream target of Transforming Growth Factor β (TGF-β) signaling [49,60,61,62]. We evaluated *Tgf-β* mRNA levels in total muscle RNA extracts from BEP and control rats and found significantly increased expression of *Tgf-β* transcripts in TA muscles but not in DIAPH (Figure 4C,F). We also observed increased levels of TGF-β3 45 kDa latent peptide in TA muscles (Figure 4A,B) but not in DIAPH (Figure 4D,E). Furthermore, we found increased expression of *Ccn2/Ctgf* mRNA in both TA and DIAPH (Figure 4G,K) from BEP-treated rats. Then, we evaluated CCN2/CTGF protein levels by western blot, and we found increased levels in the described 37 kDa band for CCN2/CTGF in TA (Figure 4H,I). In addition, we found significantly increased levels of a 50 kDa CCN2/CTGF band in BEP-treated rats compared to control animals, in both TA (Figure 4H,J) and DIAPH (Figure 4L,N). Immunostaining of transversal cryosections of TA muscle also indicated increased levels of CCN2/CTGF from BEP-treated rats (Figure 4O,P).

These results showed that episodic BEP induced a fibrotic process in skeletal muscle, which was persistent two weeks after the last episode of ethanol administration and could be one of the mechanisms contributing to decreased muscle strength. Furthermore, the increased profibrotic factor CCN2-CTGF suggested a profibrotic mechanism shared with other neuromuscular diseases [36,37,55].

### 2.4. BEP-Treated Rats Exhibit Skeletal Muscle Pathological Markers

**Figure 5:**
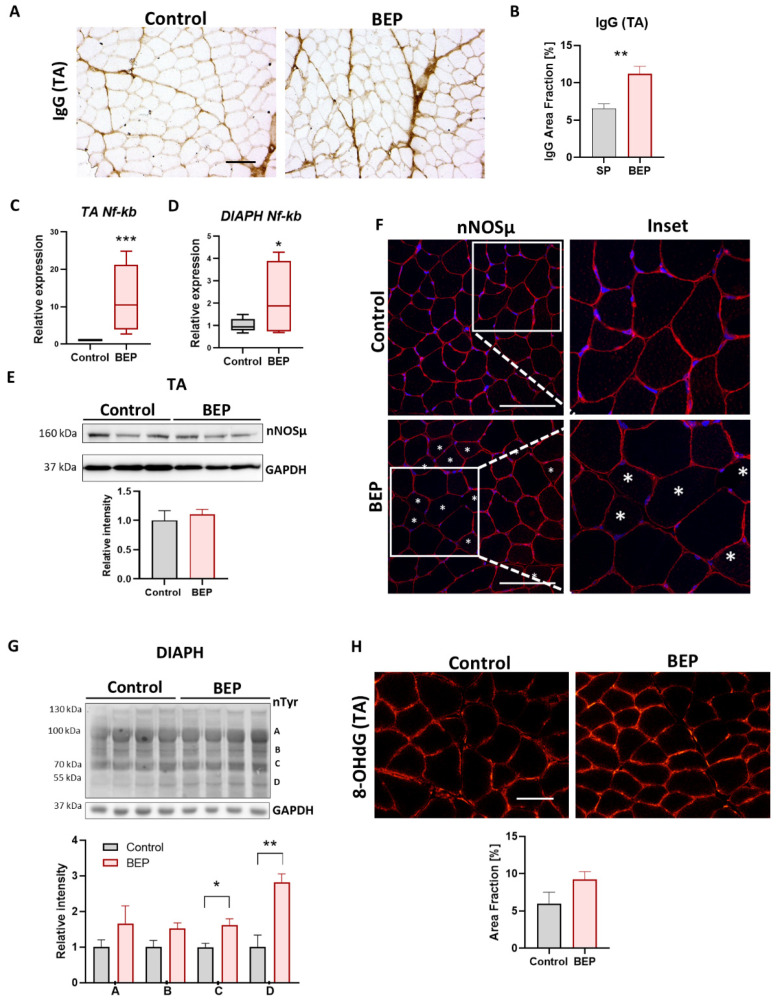
Binge-like ethanol protocol increased skeletal muscle pathological markers. (**A**) Representative images of TA immunohistochemistry using anti-Rat IgG antibody. Scale bar 100 µm. (**B**) Quantification of IgG staining as a percentage of occupied area fraction. (**C**,**D**) Relative expression of *Nf-kb* mRNA in TA ((C), control N = 3, BEP N = 4) and DIAPH ((**D**), control N = 3, BEP N = 3). (**E**) Immunoblot against nNOSµ and GAPDH as a loading control on TA muscle. Densitometric analysis performed with control N = 4, BEP N = 4. (**F**) Representative images of TA immunofluorescence using an anti-nNOSµ antibody (red) and Hoescht (blue) to stain nuclei. Asterisks indicate fibers with nNOSµ lost from the sarcolemma. Scale Bar 100 µm (**G**). Immunoblot against nTyr, with GAPDH as a loading control on DIAPH muscle and the respective densitometric analysis of bands A, B, C, D. Control N = 3, BEP N = 4. (**H**) Representative images of TA immunofluorescence using anti-8-OHdG antibody. Scale bar 100 µm. Quantification of 8-OHdG as a percentage of occupied area fraction. Control N = 3, BEP N = 4. *p*-values: * *p* ≤ 0.05; ** *p* ≤ 0.01; *** *p* ≤ 0.001.

Inflammatory markers have been previously described in skeletal muscle, in mice and rats, under chronic ethanol consumption [63,64,65,66]. Using immuno-staining of frozen cross-sections with anti-Rat IgG antibodies, we found that general skeletal muscle inflammation, evidenced by augmented rat IgG, especially around the perimysium, increased in BEP-treated rats, compared to controls (Figure 5A,B). Thus, our results suggested that episodic binge-like ethanol can trigger an inflammatory reaction that can persist over time (2 weeks after the last episode). NFκB signaling is critical for skeletal muscle homeostasis, and its aberrant activation is also involved in developing inflammation, fibrosis, and muscle waste [67,68,69,70,71,72,73]. We evaluated the *NFκB* mRNA expression, and we found increased levels in both TA and DIAPH from BEP-treated rats (Figure 5C,D).

Loss of sarcolemmal neuronal Nitric Oxide Synthase µ (nNOSμ) was observed in several neuromuscular diseases, contributing to muscle pathology [30,74,75,76,77,78,79]. Mislocalization of nNOSµ was previously suggested, together with increased oxidative stress, in one study performed with chronic ethanol-fed rats (daily ingestion for 10 weeks) [80]. However, no more research has been achieved in that area, and whether nNOSµ mislocalization occurs in other models of ethanol consumption, including binge drinking, remains unknown so far. We then evaluated nNOSµ levels and localization. We found no significant changes in nNOSµ protein levels in total extracts from TA muscle (Figure 5E). However, nNOSµ immuno-staining in cryosections showed that the sarcolemmal localization was lost in some muscle fibers from BEP-treated rats, compared to a very homogenous sarcolemmal localization in control animals (Figure 5F).

Increment of oxidative stress have been associated with skeletal muscle atrophy and tissue damage [81,82,83,84]. Oxidative stress occurs when reactive oxygen, or nitrogen, species (ROS/RNS) are increased, or when the antioxidant machine is altered, leading to accumulation of ROS/RNS in DNA, proteins and lipids [81]. We evaluated protein nitration levels on TA muscles and found increased levels of protein nitration, indicative of nitrosative stress, in BEP-treated rats, compared with control animals (Figure 5G). By immuno-staining of TA cryosections we also evaluated the presence of 8-hydroxy-guanidine (8-OhdG), an oxidized nucleoside used as a marker of oxidative damage on nucleic acids [85,86]. We observed the increased presence of this marker in BEP-treated rats, compared with control animals (Figure 5H).

The results exposed in this section suggested that episodic binge-like ethanol administration induced pathological markers related to inflammation and oxidative stress, which are also common to different neuromuscular diseases.

## 3. Discussion

Although it is known that chronic ethanol consumption can adversely affect skeletal muscle and lead to alcoholic myopathy [19,21], the actual deficit in physiological characteristics and contractile properties of whole muscles due to ethanol consumption/administration has only very recently been studied in animal models.

A study in 2019 in mice concluded that only chronic, and not binge-like ethanol consumption (one dose of 3 g/kg), decreased contractile function, measured as normalized tetanic force and fatigue in isolated EDL muscle measured 1h after ethanol administration [24]. One limitation of the mentioned study was the measure of muscle function in isolated muscle, which did not consider the critical physiological environment of the tissue, such as vascularization, oxygenation, and the contribution of the motor neuron and neuromuscular junction (NMJ) synaptic transmission, which might be affected by the presence of ethanol [87]. More recently, Laudato et al. compared male and female mice for 1 h and 24 h after administering a binge-like dose of ethanol (5 g/kg) [25]. They used an in situ approximation, which better maintained the physiological muscle environment, and they found that both males and females exposed to binge ethanol exhibited a reduction in force production and enhanced muscle fatigue 1h after ethanol administration; however, only females recovered after 24 h of ethanol clearance, while males still presented some contractile deficits [25]. Nevertheless, and despite the possible differences between rats and mice, in both mentioned studies the binge protocol consisted of a single exposure, and contractile function was measured between 1 to 24 h after ethanol administration [24,25]. In humans, muscle function has been measured immediately after a single self-reported heavy ethanol ingestion [88]. Therefore, molecular alterations that could contribute to functional muscle deficits, and that might require more time to manifest, would be undetected with these approximations.

Furthermore, the intermittent BD observed in young people and sports people was not addressed in the above-mentioned studies or others. Consequently, whether episodic BD can lead to contractile deficits has not been addressed to date. In the present work, we evaluated episodic BD, focusing on long-lasting effects on skeletal muscle. These observations were performed two weeks after the last event of ethanol ingestion, which ruled out the effects of circulating ethanol and suggested cellular and molecular changes that persisted over time. Two weeks is the average time needed for full muscle recovery and regeneration after damage [89], so is a reasonable starting point for evaluating effects that persist after alcohol clearance. Nevertheless, more research is needed to evaluate if muscle dysfunction remains longer, or if previous BD predisposes to future muscular disease. Our results showed that repetitive and intermittent binge-like ethanol administration could reduce muscle strength and increase muscle fatigability that lasted at least two weeks after the last binge episode. However, a limitation of our study resided was that only young male rats were evaluated, and further studies should be performed to contribute to the knowledge of the effects of binge alcohol in the skeletal muscle of females, which has been poorly explored, as well as the consequences in older individuals.

Compared to controls, in muscles from BEP-treated rats we observed muscle atrophy, evidenced by decreased fiber size (minimum Feret’s diameter), which was accompanied by increased levels of *atrogin-1* and *Murf-1* mRNA. It was previously shown that *atrogin-1* and *Murf-1* mRNA increase after acute ethanol administration in rats, without an immediate increase in proteolysis [90]. However, in mice, an acute dose of ethanol (binge dose) increases *atrogin-1* mRNA, but no *Murf-1* mRNA [91]. A study on female mice showed that 0–24 h after an acute binge, *atrogin-1*, and *Murf-1* mRNA results elevated, but 24–48 h post-binge *Murf-1* mRNA levels decreased [92]. On the other hand, chronic ethanol increased *atrogin-1* and *Murf-1* gene expression, which came before skeletal muscle atrophy and then returned to baseline levels when atrophy was established [93,94]. Thus, Atrogin-1 and Murf-1 mRNA and protein levels might not always directly relate to the degree of muscle atrophy observed, an observation also made in models for other diseases [95], and their levels probably depend on the timing of the evaluation after ethanol consumption.

Evidence of fibrosis through the accumulation of ECM proteins was previously shown in chronic alcohol-fed mice, rats, and macaques [63,64,66]. In this work, we showed that episodic binge-like administration of ethanol in young rats led to a fibrotic process, which was observed in the exacerbated accumulation of ECM proteins (fibronectin, collagen), increased Tgf-β (mRNA and protein) and increased profibrotic and proinflammatory factor CCN2/CTGF at mRNA and protein levels. This matricellular protein (CCN2/CTGF) is a good candidate for therapeutic approaches because it contributes to skeletal muscle fibrosis in different pathologies, and its blockage has been helpful in preventing and reducing muscle damage in the context of these pathologies [55,60,96,97]. However, to date and to our knowledge, CCN2/CTGF has not been previously studied in alcohol-related skeletal muscle pathology, nor in chronic, acute, or binge-alcohol consumption/administration patterns, although it has been shown to be increased in the liver [98]. The present work showed that BEP-treated rats had increased CCN2/CTGF at protein levels, evidenced by two main immunoreactive bands in western blot assays, the known 37 kDa and a bigger band of 50 kDa, which both increased due to BEP treatment. Previous studies detected the presence of this 50 kD band, which was associated with the formation of dimers of CCN2/CTGF proteolytic fragments [99], and proteolytic fragments are suggested to be essential for CCN2/CTGF biological activity [100].

*Ccn2/Ctgf* is a target gene for TGF-β signaling, one of the main and most studied profibrotic routes in skeletal muscle and other tissues [50,53,101,102,103] and we found increased levels of Tgf-β, mRNA and protein. Nevertheless, CCN2/CTGF can also be induced by other signaling pathways, working independently or simultaneously with TGF-β, which are involved in pathological conditions and might also be present in alcohol muscle damage, such as hypoxia (through HIF-1α) [104,105], lysophosphatidic acid [106,107,108], and YAP/TAZ signaling [109,110]. YAP has been found activated in the liver of patients with a history of alcohol abuse, and it is also activated in the livers of chronic/binge alcohol-treated mice, associated with increased CCN2/CTGF [98], although whether this happens in skeletal muscle remains unknown. Chronic and binge alcohol has been shown to increase hypoxia and activate hypoxic response through HIF-1α in the liver [98,111,112,113,114] and the pancreas [115,116], where it is suggested to have a role in contributing to alcoholic-related diseases in those organs [117]. However, whether hypoxia and HIF-1α activation occurs in skeletal muscle and contributes to increasing CCN2/CTGF as a consequence of acute, chronic, or binge ethanol consumption/administration, is a matter that remains to be studied.

Therefore, other signaling pathways involved in the atrophic and fibrotic processes might be involved in the skeletal muscle response to ethanol in different consumption patterns remain to be studied. For instance, NFκB signaling is critical for skeletal muscle homeostasis, and its aberrant activation is also involved in developing inflammation and fibrosis in muscular dystrophies [67,68,69]. Furthermore, NFκB is known to cause muscle wasting and atrophy in different pathological conditions [70,71,72,73]. However, NFκB has not been studied before in alcohol-related skeletal muscle pathology; even though we only evaluated mRNA levels in BEP-treated rats. A future investigation could reveal NFκB signaling involvement in the skeletal muscle consequences of different patterns of problematic alcohol consumption. As another example, we observed loss of sarcolemmal nNOSµ in some muscle fibers from BEP-treated rats. Loss of nNOSμ from the sarcolemma is observed in several neuromuscular diseases, contributing to muscle pathology by decreasing oxygen availability but also by increasing nitrosative stress as a toxic gain-of-function when cytosolic NO, instead of sarcolemmal, is produced, leading to hypernitrosylation of proteins, and the activation of both atrophic and fibrotic pathways [30,74,75,76,77,78,79]. Furthermore, loss of nNOSµ leads to intensified fatigue in vivo [78] and in situ after repeated tetanic stimulations [118], while restoring sarcolemmal nNOSµ or increasing NO-signaling can decrease muscle fatigue [30,78,119]. Further investigation is needed to evaluate the nature of the fibers missing sarcolemmal nNOSµ, and its role in skeletal muscle dysfunction observed in our binge-like ethanol administration protocol.

### Summary and Perspectives

Binge episodic ethanol consumption is highly prevalent in young people, and amateur and professional athletes. Pathological mechanisms have been previously described as associated with alcoholic myopathy, which occurs in chronic alcohol consumers; however, the effects of episodic BD on skeletal muscle function are less characterized, and less information contributes to a low-risk perception of the long-term consequences of BD. 

We used an intermittent binge-like ethanol protocol (BEP) in young rats as a model of episodic BD, and we evaluated muscle function and pathological markers two weeks after the last ethanol administration. Our results showed that BEP led to decreased maximal force and enhanced fatigability in a hindlimb muscle. Furthermore, the deficit in contractile properties was accompanied by muscle atrophy, and the presence of a fibrotic process which was evidenced by increased profibrotic factor CCN2/CTGF and accumulation of ECM proteins. Other features, such as increased endogenous IgG, NFκB expression, oxidative stress, and loss of nNOSµ in some muscle fibers, suggested that other signaling pathways, common to several neuromuscular diseases, might contribute to muscle dysfunction under episodic binge-like ethanol administration. This work contributes to the knowledge that episodic binge drinking causes detrimental processes in skeletal muscle, affecting muscle function. In the context of BD behavior in youth and people related to sports, these observations might contribute to elevating the risk perception about BD’s negative consequences in athletic performance.

## 4. Materials and Methods

### 4.1. Binge-like Ethanol Protocol in Rats

Postnatal day 25 (PND 25) male Sprague Dawley rats were housed in groups of 3–4 rats per cage and maintained at 22 °C on a 12:12 h light-dark cycle, with food and water ad libitum. The rats were randomly assigned to the control (saline solution) or the Binge-like Ethanol Protocol (BEP, 3.0 g/kg, 25% *v/v* in isotonic saline solution). Solutions were administered by intraperitoneal injections (IP) beginning on PND25 as previously described [26,120,121]. A second dose was administrated on PND26, followed by two consecutive days without ethanol administration, a process repeated four times. Specifically, animals received ethanol administration at PND25, 26, 29, 30, 33, 34, and 37, 38. The injected solution volume depended on each animal’s weight to reach the desired dose. In this protocol, a dose of ethanol the maximum blood ethanol concentrations (BEC) reached 210 ± 11 mg/dL at 30 min post-injection, followed by a gradual decline in the next hours [26,29]. Two weeks after the last IP ethanol administration, muscle contractile properties were analyzed; then, animals were euthanized by decapitation. Muscle samples for cryosectioning were frozen in liquid nitrogen cooled-isopentane (Merk) and stored at −80 °C until processing.

### 4.2. In Situ Analysis of Muscle Contractile Properties

Muscle strength was determined as previously described [30] Isofluorane anesthetized rats (3.0% isoflurane gas in pure oxygen) were placed on a 37 °C heated platform. The knee was restrained with a surgical needle, and the distal tendon of the TA was surgically isolated and attached to a force transducer (UFI, Morro Bay, CA, USA). The TA muscles were activated by stimulation of the sciatic nerve using two electrodes. TA was adjusted to an optimum length (L_0_) to produce the maximum tetanic force. While held at Lo, the TA was stimulated every 1 min at increasing frequencies (1 to 200 Hz) to generate force-frequency curves. TA muscles were subjected to repeated isometric stimulations (150 Hz) at 2,5-s intervals for 3 min to test resistance to exercise-induced fatigue. Fatigue recovery was recorded every 1 min for 5 min. The specific force was calculated by normalizing net force to the physiological cross-sectional area (L_0_×density [1.06 g/mm^3^]/muscle mass).

### 4.3. Immunohistochemistry

Muscle cryosections (10 µm) were fixed in cold ethanol, and then in RT methanol with 0.3% H_2_O_2_). Samples were washed in PBS and incubated overnight with primary anti-Rat IgG antibody (A21093, Invitrogen, Waltham, MA, USA, 1:100) in blocking solution (PBS, 0.25% Triton X-100). Sections were placed at RT°, washed in PBS, and then incubated with HRP-conjugated secondary antibodies (1:500) for 1 h. The immunoperoxidase reaction was visualized after incubation in 0.1% diaminobenzidine, 0.03% H_2_O_2_ [122]. Sections were washed with tap water, dehydrated in an ethanol gradient, cleared with xylene, and mounted with a mounting medium (Eukitt, Sigma Aldrich, St. Louis, MO, USA). Cross-sections were visualized on a Leica DM2000 using Mshot Image Analysis System software with 10× objectives

### 4.4. Indirect Immunofluorescence

For TA, DIAPH, and EDL immunofluorescence, cryosections of 10 µm were fixed in 4% paraformaldehyde (Winkler), permeabilized with PBS-0.05% Triton, and blocked for 1 h with blocking buffer (BSA 2%, 0.05% Triton X-100 in PBS). Samples were incubated overnight at 4 °C with: anti-Fibronectin (F3648, Sigma Aldrich), anti-Collagen I (PA1-26204, Invitrogen, Waltham, MA, USA), anti-Slow Myosin (M8421, Sigma Aldrich), anti- nNOSµ (617000, Invitrogen) and anti-IIA myosin heavy chain antibody (SC-71, DSHB, University of Iowa, Iowa City, IA, USA). The corresponding Alexa Fluor- 568 or 488-conjugated anti-IgGs (Invitrogen) were used as secondary antibodies. In addition, fluorescent wheat germ agglutinin (WGA) (Thermo Fisher, Waltham, MA, USA) was used to label cell membranes and Hoescht for nuclear staining. Slices were washed and mounted in Fluoromount G. Samples were visualized on a Nikon Eclipse E600 using NIS Elements software v4.20 or Leica DM2000 epifluorescence using Mshot Image Analysis System software with 20× or 40× objectives.

### 4.5. Sirius Red Staining

TA, DIAPH, and soleus cryosections (10 µm) were fixed in 100% ethanol, total and fibrillar collagen content was detected by staining with 1% Sirius red in picric acid, as previously described [123,124]. Samples were visualized on a Nikon Eclipse E600 using NIS Elements software v4.20 or Leica DM2000 using Mshot Image Analysis System software with 10× or 20× objectives.

### 4.6. Determination of Occupied Area and Fiber Diameter

To quantify the percentage of the occupied area by Fibronectin, Collagen I, Sirius red, and IgG, microphotographs from transversal muscle cryosections color threshold were adjusted, and the area percentage was measured with ImageJ v1.53k software (NIH). Muscle fiber size was evaluated by determination of minimal Feret’s diameter in WGA stained sliced using the ROI manager plugin from ImageJ software. Quantifications were performed using 4–7 images per muscle at 10× magnification.

### 4.7. Determination of Fiber Type Percentage

To quantify the percentage of slow type I and fast II fibers, microphotographs from transversal muscle cryosections were immuno-stained with anti-Slow Myosin (M8421, Sigma Aldrich) and Alexa Fluor-488 conjugated WGA. Type IIA fibers were immuno-stained with anti-IIA myosin heavy chain antibody (SC-71, DSHB) and Alexa Fluor-488 conjugated WGA. Fast (not stained) and slow type I fibers were counted using the multipoint tool plugin from ImageJ software. The fiber type percentage was calculated using individual images of TA, and DIAPH. Quantifications were performed using 4–7 images per muscle at 20× magnification.

### 4.8. Western Blot

Skeletal muscles were homogenized in one volume of Tris-EDTA buffer pH 7.4 with 1 Mm phenylmethyl-sulfonyl fluoride (PMSF) using Ultraturrax T25 (Janke & Kunkel IKA Werk, Staufen, Germany). Then, the same volume of buffer containing 2% glycerol, 4% SDS, and 0.125 M Tris pH 6.8 was added to the homogenates and mixed. Muscle homogenates were incubated at 50 °C for 20 min and centrifuged at 14,000 rpm to separate insoluble material. Protein supernatant content was determined using BCA Assay Kit (Thermo Fisher) with BSA as the standard. Muscle extract aliquots (20–40 µg) were subjected to SDS-PAGE and transferred onto PVDF membranes (Thermo Fisher). Membranes were blocked in 5% BSA in TBS (50 Mm Tris-CL, pH 7.6; 150 Mm NaCl) and then incubated at 4 °C overnight with: anti-Fibronectin (F3648, Sigma Aldrich), anti-nTyr (N0409, Sigma), anti-Atrogin-1 (PA5-106917, Invitrogen), anti-CTGF (D8Z8U, Cell Signaling, Danvers, MA, USA), anti-GAPDH (631402, Biolegend, San Diego, CA, USA), anti-nNOSµ (617000, Invitrogen), anti- TGF-β3 (D-B3, DSHB). Membranes were incubated with HRP-conjugated secondary antibodies and visualized by enhanced chemiluminescence (Cyanagen, Bologna, Italy) in ImageQuant LAS 500 equipment. Densitometric analysis and quantification were performed using ImageJ software (NIH).

### 4.9. RNA Isolation, Reverse Transcription, and Quantitative Real-Time PCR

Total RNA was isolated from TA and DIAPH muscle using TRIzol (Invitrogen), according to the manufacturer’s recommendations. Total mRNA was reverse transcribed into cDNA using iScript RT Supermix containing oligo (dT) primers and M-MLV reverse transcriptase (1708841, BioRad, Hercules, CA, USA). Quantitative real-time PCR reactions were performed in triplicate using PowerUp SYBR Green Master Mix (Applied Biosystems, Waltham, MA, USA) on an Agilent AriaMx Real-Time PCR System (Agilent Technologies). mRNA expression was quantified with the comparative ΔCt method (2^−ΔΔCt^), using 18S as the reference gene. mRNA levels were expressed relative to the mean expression in control rats. Primers set used: *Atrogin-1* (Fwd: 5′-TACTAAGGAGCGCCATGGATACT-3′; Rev: 5′-GTTGAATCTTCTGGTATCCAGGAT-3′), rat *Murf-1* (Fwd: 5′-GGTGCCTACTTGCTCCTTGTGC-3′; Rev: 5′-AGTCTGAACTCGGTCGTTCCCT-3′), rat *Ccn2* (Fwd: 5′-AATGCTGTGAGGAGTGGGTGT-3′; Rev: 5′-GTTGGCTCGCATCATAGTTGG-3′), rat *Tgf-β1* (Fwd: 5′-CAACGCAATCTATGACAAAACC-3′; Rev: 5′-ACAAGAGCAGTGAGCACTGAAG-3′), rat *Nf-kB* (Fwd: 5′-ATGTGGAGATCATTGAGCAGC-3′; Rev: 5′-CCTGGTCCTGTGTAGCCATT-3′), and rat *18S* (Fwd: 5′-GTAACCCGTTGAACCCCATT-3′; Rev: 5′-CCATCCAATCGGTAGTAGGC-3′).

### 4.10. Hematoxilin & Eosin Staining

Muscle cryosections (10 μm) were placed onto glass slides. Hematoxylin and eosin (H&E) staining was performed to assess muscle architecture and possible infiltration. Briefly, tissue sections were fixed in 4% paraformaldehyde, washed with tap water, incubated for 5 min with diluted H&E (Merck, Rahway, NJ, USA; 25% *v/v* in H_2_O), followed by a wash with tap water. Eosin (1%, Merck) was added for 30 s and then samples were dehydrated with increasing concentrations of ethanol. Finally, Entellan (Merck) was added for sliced mounting. Sections were imaged using bright field microscopy on a Nikon Eclipse E600 using NIS Elements software v4.20. We obtained 4–7 images per muscle at 20× magnification.

### 4.11. Statistical Analyses

Data and statistical analyses were performed using Prism8 software (Graph Pad 8 Software). Data is presented as Mean ± SEM or Box and Whiskers graphs. When analyzing two groups, differences we analyzed with Welch’s T-test. More than two groups were analyzed using one-way or two-way ANOVA according to the number of variables. Sidak’s multiple comparison test was performed to compare differences between groups unless noted differently. Symbology for *p*-values: * *p* ≤ 0.05; ** *p* ≤ 0.01; *** *p* ≤ 0.001; **** *p* ≤ 0.0001.

## Data Availability

All raw data supporting the conclusions of this article are available upon request. Please contact with the corresponding author of the article.

## Supplementary Materials

The following supporting information can be downloaded at: https://www.mdpi.com/article/10.3390/ijms24021655/s1.
