# Supplementary material for: Episodic Binge-like Ethanol Reduces Skeletal Muscle Strength Associated with Atrophy, Fibrosis, and Inflammation in Young Rats
Source: Int J Mol Sci. 2023 Jan 14;24(2):1655. doi: 10.3390/ijms24021655 (PMC9861047; doi:10.3390/ijms24021655)
Supplement: Supplementary file 1 [file ijms-24-01655-s001.zip › ijms-2027841-supplementary.pdf]

International Journal of *Molecular Sciences*

Supplementary Figures

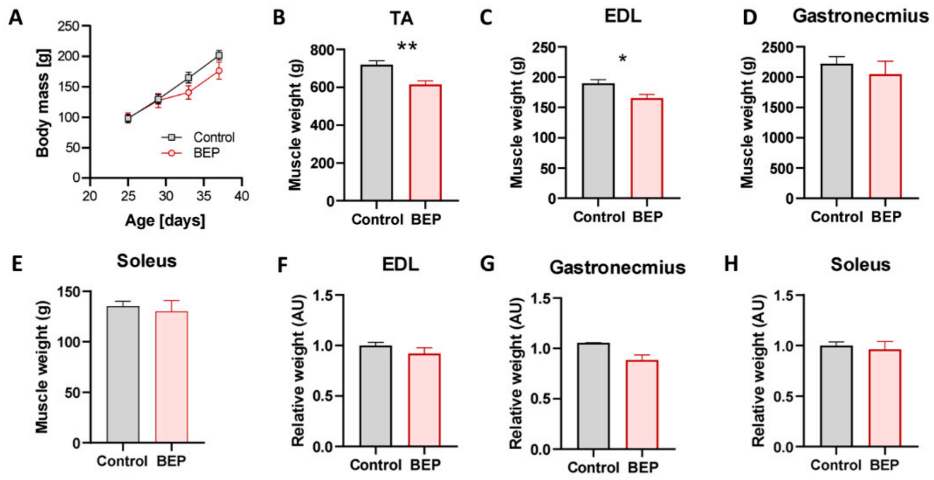

Supplementary Figure S1. Binge-like ethanol protocol decreases muscles weight. Muscle weight of TA (A), EDL (B), Gastrocnemius (C), and soleus (D). **E-G**. The relative weight of EDL, Gastrocnemius (F), and soleus (G). Relative weight was determined by normalizing the weight of muscles to the weight of control muscles. Graphs represent mean ± SEM. N Control = 2 N BEP = 4. * $p \leq 0.05$; ** $p \leq 0.01$.

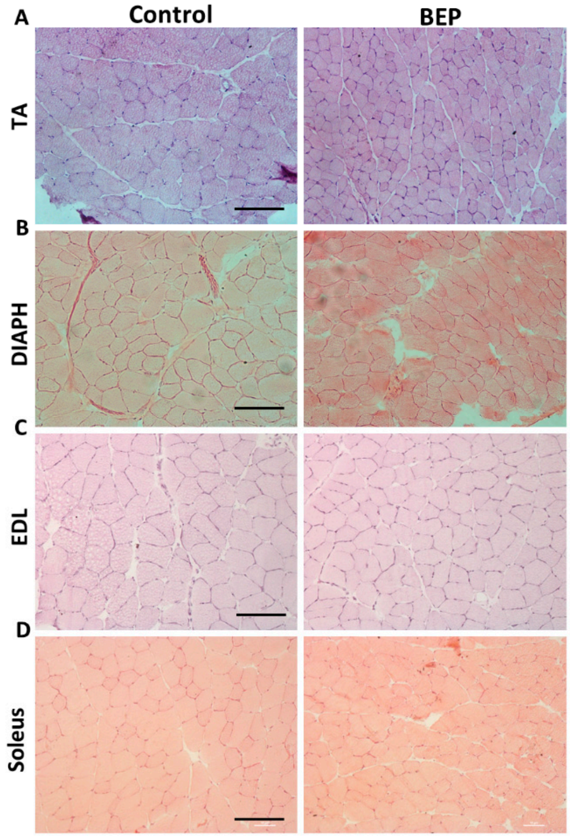

Supplementary Figure S2. Binge-like ethanol protocol does not alter muscle morphology. A, B, C and D. Representative images of hematoxilyn eosin stained muscle sections of TA (A), DIAPH (B), EDL (C) and Soleus (D). Scale bar 100. Control N= 6, BEP N= 4.

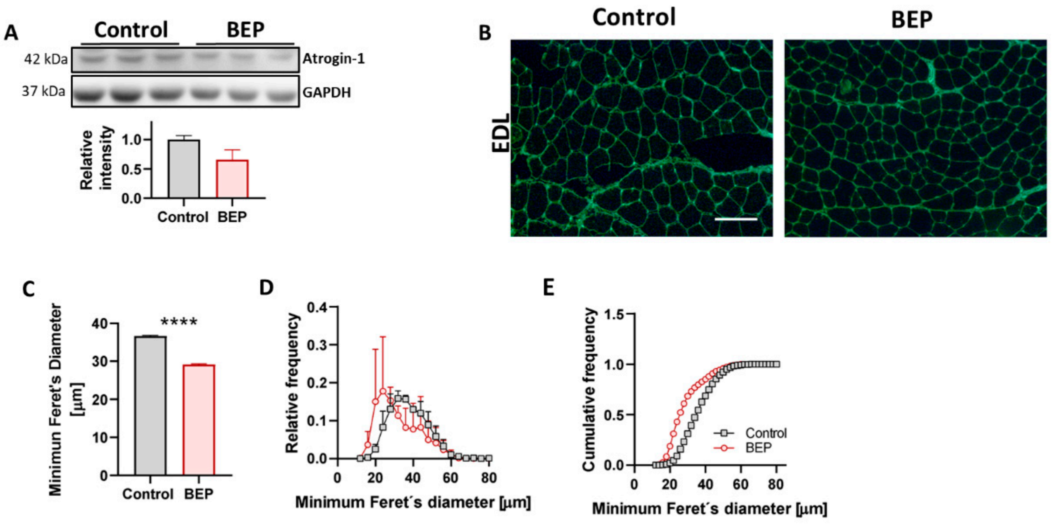

Supplementary Figure S3. Binge-like ethanol protocol induces skeletal muscle atrophy. **A.** Immunoblot of Atrogin-1, and GAPDH as a loading control, and densitometric quantification on TA muscle. Control N=4, BEP N=4. **B.** Representative image of WGA-stained muscle sections of EDL. Scale bar 100 µm. **C.** Quantification of minimum Feret's diameter of skeletal muscle fibers of EDL. Control N=3, BEP N=2. Graphs represent mean ± SEM, 7 images per N. **D.** Histogram showing relative frequencies of minimum Feret's diameter in EDL. **E.** Histogram showing cumulative frequencies of minimum Feret's diameter in EDL. Graphs represent mean ± SEM. **** $p \leq 0.0001$.

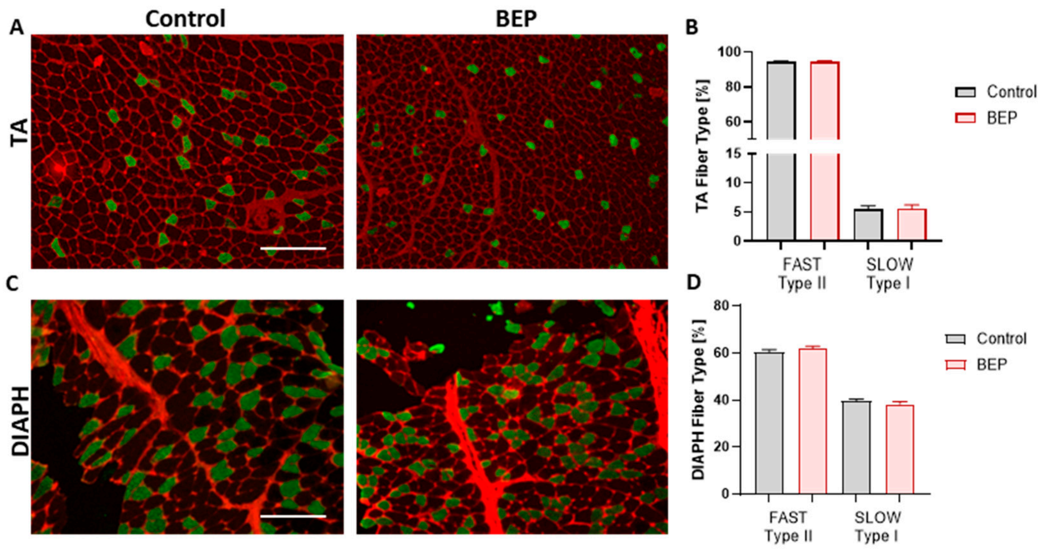

Supplementary Figure S4. Binge-like ethanol protocol does not alter muscle fiber type proportion. A, C. Representative images of TA (A) and DIAPH (C) immunofluorescence using WGA (red) and anti- Slow Myosin antibody (green). Fibers stained in green correspond to slow type I fibers, and non-stained fibers in black correspond to fast type II fibers. Scale bar:100 µm. **B, D.** Percentage of fiber types on TA (B) and DIAPH (D). Control N=5, BEP N=6.

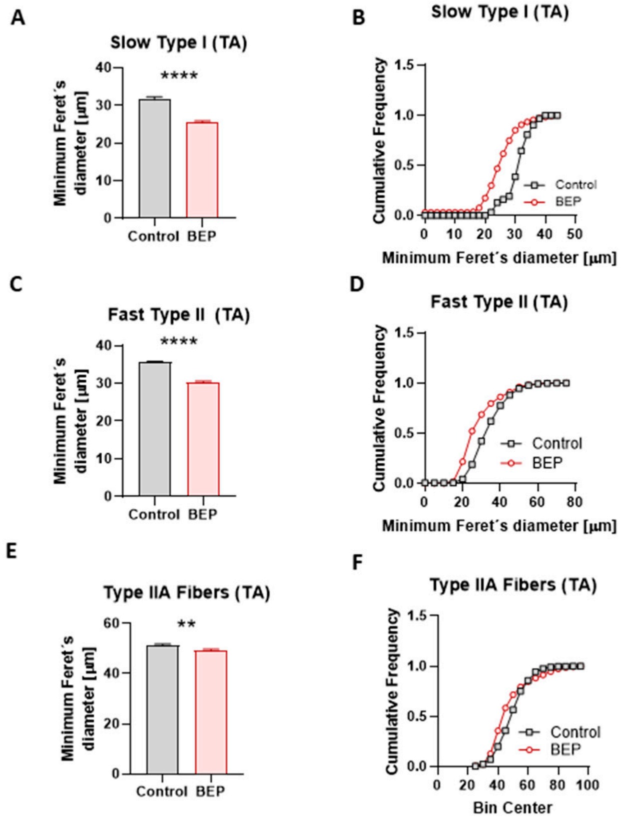

Supplementary Figure S5. Binge-like ethanol protocol induce atrophy independent of skeletal muscle fiber type. **A, C, E.** Quantification of minimum Feret's diameter of slow type I (A), fast II type (C) and type IIa (E) skeletal muscle fibers of TA. Control N=3, BEP N=2. **F, I.** Histograms showing cumulative frequencies as fraction of minimum Feret's diameter of slow type I (B), fast II type (D) and type IIa (F) skeletal muscle fibers of TA fast (F) and slow (I) skeletal muscle fibers of TA. Control N=3, BEP N=2. ** $p \leq 0.01$; **** $p \leq 0.0001$

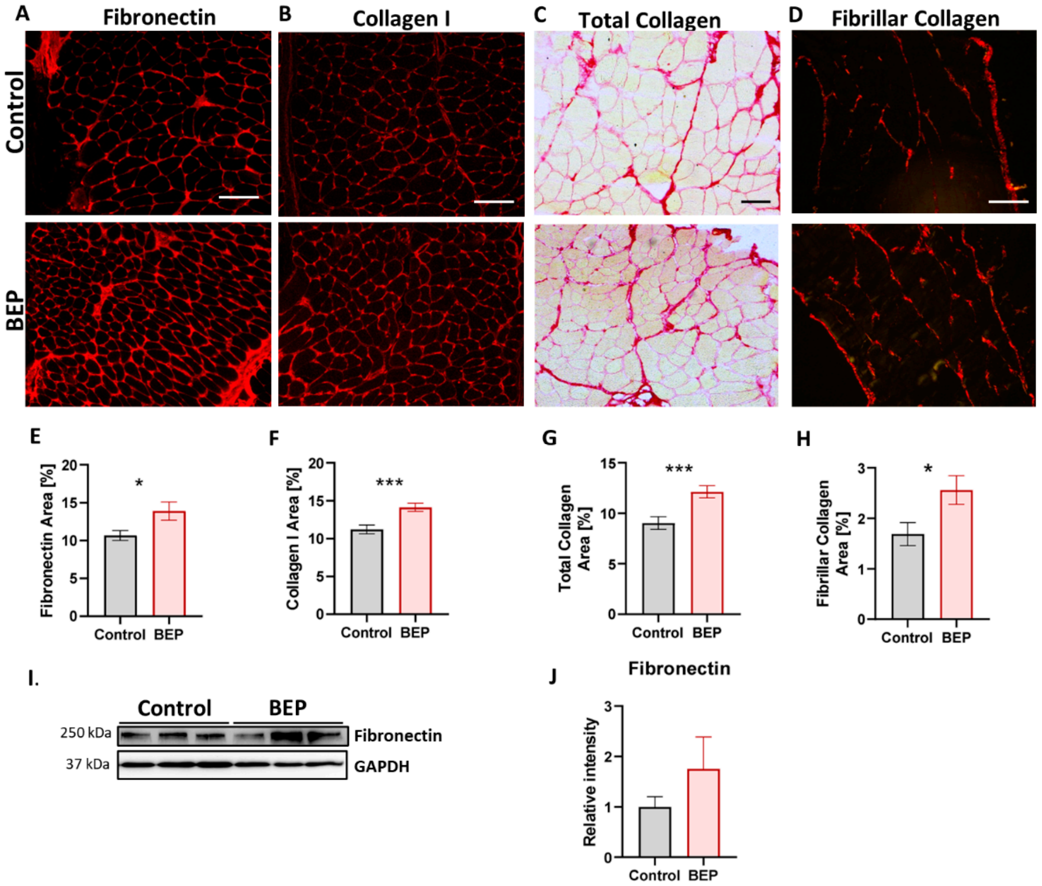

Supplementary Figure S6. Binge-like ethanol protocol induces ECM protein accumulation and enhances fibrotic markers on DIAPH muscle. **A, B**. Representative images of DIAPH immunofluorescence using anti- fibronectin (A) and anti-collagen I (B) antibodies. **C, D**. Representative images of Sirius Red staining in brightfield microscopy showing total collagen (C) and polarized light microscopy showing fibrillar collagen (D). Scale bar 100 µm. **E-H**. Quantification of fibronectin (E), collagen I (F), total collagen (G), and fibrillar collagen (H) as a percentage of occupied area fraction. Control N=5, BEP N=6. **I.** Immunoblot against fibronectin and GAPDH as a loading control. **J.** Densitometric quantification of immunoblots against fibronectin. Control N=4, BEP N=4. * $p \leq 0.05$; *** $p \leq 0.001$.

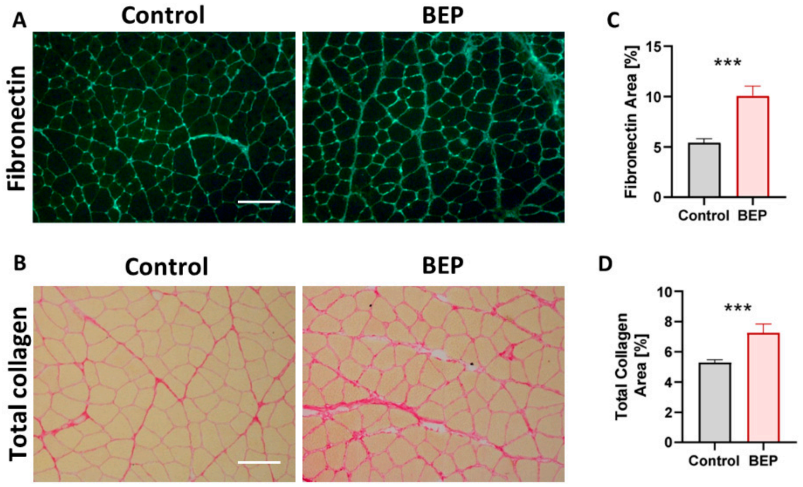

Supplementary Figure S7. Binge-like ethanol protocol induces ECM protein accumulation on EDL and soleus muscle. **A.** Representative images of EDL immunofluorescence using anti-fibronectin antibody. **B**. Representative images of Sirius Red staining in brightfield microscopy showing total collagen in soleus. **C, D**. Quantification of fibronectin (C) and total collagen (D) as a percentage of occupied area fraction. Control N=3, BEP N=2. Graphs represent mean ± SEM, 7 images per N. Scale bar 100 µm. *** $p \leq 0.001$.
